# Prevalence of rifampicin resistance in pulmonary tuberculosis: A laboratory-based study

**DOI:** 10.1371/journal.pgph.0005525

**Published:** 2025-12-01

**Authors:** Israel Bedzina, Agnes Naa Larley Lartey, Lois Kwaley-Buabeng, Prosper Mensah, Gameli Nudo, Eric Selorm Odum, Rita Sewornu, Kenneth Ablordey, John Gameli Deku, Kwabena Obeng Duedu

**Affiliations:** 1 Fly Zipline Ghana Limited, Kete-Krachi, Oti Region, Ghana; 2 Department of Medical Laboratory Sciences, School of Allied Health Sciences, University of Health and Allied Sciences, Ho, Ghana; 3 Laboratory Department, Ho Teaching Hospital, Ho, Ghana; 4 College of Life Sciences, Faculty of Health, Education and Life Sciences, Birmingham City University, Birmingham, United Kingdom; Faculty of Medicine, Khon Kaen University, THAILAND

## Abstract

Tuberculosis (TB) remains a significant global public health challenge, particularly in low- and middle-income countries. The emergence of rifampicin-resistant tuberculosis further complicates control efforts. Despite national efforts, there are limited data on the prevalence of *Mycobacterium tuberculosis* and their rifampicin resistance in Ghana, especially at the facility level. This study aimed to determine the prevalence of *Mycobacterium tuberculosis* and rifampicin resistance among presumptive tuberculosis cases at the Ho Teaching Hospital over a three-year period. The study used a retrospective design and collected secondary data from the Microbiology Laboratory Unit of Ho Teaching Hospital between 2022 and 2024. Data on patient demographics, *Mycobacterium tuberculosis* detection, and rifampicin resistance were retrieved and analysed using descriptive and inferential statistics. *Mycobacterium tuberculosis* and rifampicin resistance were identified using the GeneXpert MTB/RIF assay. Statistical analyses were performed using STATA version 15 with statistical significance set at p < 0.05. Out of 2,225 presumptive tuberculosis cases, 203 tested positive for the infection, resulting in an overall prevalence of 9.1% (95% CI: 7.9–10.4). The prevalence was significantly higher among males (12.4%) compared to females (5.7%), and highest among young adults aged 18–24 years (12.8%). Of the 166 *Mycobacterium tuberculosis-*positive cases tested for rifampicin susceptibility, 3 (1.8%) were resistant. Although rifampicin resistance was more common among females and adults, the differences were not statistically significant. Although the detection rate of rifampicin resistance among newly diagnosed *Mycobacterium tuberculosis* cases was low (1.8%), it remains a significant public health concern, hence the need for enhancing surveillance systems and prioritising early detection strategies.

## Introduction

*Mycobacterium tuberculosis* is the bacterium that causes tuberculosis (TB) and is a highly adaptable pathogen with significant genetic diversity that influences disease severity and immune response [1]. *Mycobacterium tuberculosis* primarily infects the lungs but can disseminate to other parts of the body through mechanisms involving infected alveolar macrophages, which migrate and facilitate bacterial spread [2]. During infection, the bacterium persists in granulomas, using effector proteins to evade immunity, maintain latency, and contribute to pulmonary or systemic disease [3].

The global prevalence of tuberculosis in 2023 is estimated by the World Health Organisation (WHO) to involve about 10.8 million new cases, equivalent to 134 incident cases per 100,000 population. The burden is highly concentrated in eight countries, led by India (26%), followed by Indonesia, China, the Philippines, Pakistan, Nigeria, Bangladesh, and the Democratic Republic of the Congo [4]. Incidence varies widely by country, from less than 10 to more than 500 cases per 100,000 population annually, with the highest rates typically in African countries [4]. Tuberculosis affects all age groups but predominantly adult men, who represent 55% of cases, with women constituting 33% and children 12%. Detection rates of new tuberculosis cases overall reach about 75% but are lower in children, especially those under 5 years old [4]. In the case of Ghana, 43 cases were detected from 2015 to 2019 in the Sene East district during surveillance [5]. A recent study in a teaching hospital by Deku, Aninagyei [6] reported a TB prevalence of 10.76% and rifampicin-resistant tuberculosis (RR-TB) prevalence of 3.45%.

Epidemiological data from various settings confirm the continued presence of RR-TB and highlight the prevalence of monoresistance and multidrug resistance, often higher in previously treated patients and those with comorbidities such as diabetes. These findings underline the importance of early detection and targeted interventions to prevent spread and improve treatment success [7]. In addition, there is a paucity of data on rifampicin-resistant tuberculosis. Hence, this study was designed to assess the prevalence of *Mycobacterium tuberculosis* and rifampicin resistance using GeneXpert data over a 3-year period (2022–2024) at the Ho Teaching Hospital of Ghana.

## Methodology

### Study design

The study utilised a retrospective study design, which analysed secondary data collected on tuberculosis from the Microbiology Laboratory Unit of the Ho Teaching Hospital. The data analysed in this study were archived from 2022 to 2024.

### Study site

The study was carried out at the Microbiology Laboratory Unit of Ho Teaching Hospital in the Ho Municipality, Volta Region, Ghana. This laboratory unit is responsible for processing urine, stool, sputum, and other bodily fluids for infectious pathogens. Ho, the capital city of the Volta Region, is a rapidly growing urban centre with a diverse population of approximately 180,420 people, comprising 84,843 males and 95,577 females [8]. About 62% of the population resides in urban localities, which presents a rich setting for studying health-related issues in both urban and peri-urban contexts. Geographically, Ho is situated between Mount Adaklu and Mount Galenukui. The municipality shares boundaries with Adaklu and Agotime-Ziope districts to the south, Ho West district to the north and west, and the Republic of Togo to the east. The coordinates of Ho Teaching Hospital are 6.60126^o^N 0.48404^o^E. The Hospital serves as the primary referral facility in the Volta region, catering to over 100,000 patients, with a bed capacity of 340.

### Study participants

The study participants included records of tuberculosis testing performed and archived between 2022 and 2024 at the Ho Teaching Hospital.

### Inclusion and exclusion criteria

All records of tuberculosis testing performed and archived between 2022 and 2024 were included in the study. However, tuberculosis test records with incomplete data (missing age, gender, and test results) were excluded from the study.

### Laboratory testing (GeneXpert assay)

Sputum samples with sufficient volume were tested with Xpert *Mycobacterium tuberculosis*/Rifampicin (MTB/RIF) Version 5.0 assay on the GeneXpert platform (Cepheid, USA) to detect the presence of *Mycobacterium tuberculosis* and rifampicin resistance, as per the manufacturer’s instructions.

### Data collection

Archived data from the hospital microbiology unit were collected, sorted, and entered into a Microsoft Excel 365 file and prepared for analysis. Data retrieved were age, gender, year of infection, tuberculosis test result and rifampicin resistance. The archived records were accessed and retrieved between June 23, 2025 and July 7, 2025.

### Outcome variables

The outcome variable for this study was the prevalence of *Mycobacterium tuberculosis* cases, and the prevalence of rifampicin resistance among the *Mycobacterium tuberculosis* cases over the study period.

### Independent variables

Years used in the study, age and gender of patients were independent variables in this study.

### Data analysis

Data were entered into Microsoft Excel 365, cleaned, and exported to STATA version 15 for analysis. Descriptive and inferential statistics were performed, with prevalence estimates reported at 95% confidence intervals. Differences across age, sex, year, and month were tested using chi-square. Two multivariable logistic regression models were built: Model 1 adjusted for age, year, and month, while Model 2 also included sex. Results were presented as adjusted odds ratios (aORs) and adjusted prevalence ratios (aPRs) with p-values. Model fit was assessed using Akaike Information Criterion (AIC), Bayesian Information Criterion (BIC), and Area Under the Receiver Operating Characteristic Curve (AUC). TB prevalence and rifampicin resistance were examined over time using graphs and linear regression (R²). Rifampicin resistance was further analysed with chi-square and logistic regression. A p-value <0.05 was considered statistically significant.

### Ethical consideration

Ethical approval with reference number UHAS-REC A.8 [57] 24–25 was obtained from the Research Ethics Committee of the University of Health and Allied Sciences. Additionally, written permission was obtained from the management of the Ho Teaching Hospital for the use of data generated at the microbiology unit in the facility for the study. Informed consent was not obtained from the participants since the study was a retrospective one. Data were fully anonymised before access, and no information that could be linked to the participants was retrieved. Informed consent was waived by the Research Ethics Committee of the University of Health and Allied Sciences, Ho, Ghana. All archived data for the study were kept undisclosed and used for the study only by keeping them on a password-protected computer accessible to only the principal investigator.

## Results

Among 2,225 presumptive tuberculosis cases, *Mycobacterium tuberculosis* was detected in 203 individuals, representing an overall prevalence of 9.1% (95% CI: 7.9–10.4). The prevalence varied significantly across age groups (*p* < 0.001), with the highest detection observed among young adults aged 18–24 years (12.8%; 95% CI: 8.0–19.1) and adults aged 25–64 years (11.2%; 95% CI: 9.6–13.0), while children aged 1–12 years showed the lowest rates (2.0–2.7%). A significant difference was also observed between sexes (*p* < 0.001), with males exhibiting a higher prevalence (12.4%; 95% CI: 10.5–14.5) compared to females (5.7%; 95% CI: 4.4–7.3). Table 1.

**Table 1:** Prevalence of *Mycobacterium tuberculosis* and sociodemographic factors among presumptive tuberculosis cases.

Variables	*Mycobacterium tuberculosis*			
	Overall	MTB not detected	MTB detected	P-value
	n (%)	n (%) [95% CI]	n (%) [95% CI]	
Total	2225 (100.0)	2022 (90.9) [89.6-92.1]	203 (9.1) [7.9-10.4]	
**Age (years)**				
Infants (Under 1)	54 (2.4)	50 (92.6) [82.1-97.9]	4 (7.4) [2.1-17.9]	
Toddlers and pre-schoolers (1–4)	73 (3.3)	71 (97.3) [90.5-99.7]	2 (2.7) [0.3-9.5]	
School-age children (5–12)	152 (6.8)	149 (98.0) [94.3-99.6]	3 (2.0) [0.4-5.7]	**<0.001**
Adolescents (13–17)	91 (4.1)	89 (97.8) [92.3-99.7]	2 (2.2) [0.3-7.7]	
Young adults (18–24)	156 (7.0)	136 (87.2) [80.9-92.0]	20 (12.8) [8.0-19.1]	
Adults (25–64)	1323 (59.5)	1175 (88.8) [86.9-90.5]	148 (11.2) [9.6-13.0]	
Elderly (65 and above)	376 (16.9)	352 (93.6) [90.6-95.8]	24 (6.4) [4.1-9.4]	
**Sex**				
Female	1085 (48.8)	1023 (94.3) [92.8-95.6]	62 (5.7) [4.4-7.3]	
Male	1140 (51.2)	999 (87.6) [85.5-89.5]	141 (12.4) [10.5-14.5]	**<0.001**
**Year**				
2022	681 (30.6)	608 (89.3) [86.7-91.5]	73 (10.7) [8.5-13.3]	
2023	797 (35.8)	721 (90.5) [88.2-92.4]	76 (9.5) [7.6-11.8]	0.064
2024	747 (33.6)	693 (92.8) [90.7-94.5]	54 (7.2) [5.4-9.3]	
**Months**				
January	205 (9.2)	180 (87.8) [82.5-91.9]	25 (12.2) [8.0-17.5]	
February	192 (8.6)	175 (91.2) [86.3-94.8]	17 (8.8) [5.2-13.7]	
March	196 (8.8)	182 (92.9) [88.4-96.1]	14 (7.1) [3.9-11.6]	
April	184 (8.3)	171 (92.9) [88.2-96.2]	13 (7.1) [3.4-11.8]	
May	193 (8.7)	173 (89.6) [84.4-93.5]	20 (10.4) [6.5-15.6]	
June	186 (8.4)	174 (93.6) [89.1-96.7]	12 (6.5) [3.4-11.1]	0.220
July	162 (7.3)	149 (92.0) [86.7-95.7]	13 (8.0) [4.3-13.3]	
August	210 (9.4)	190 (90.5) [85.7-94.1]	20 (9.5) [5.9-14.3]	
September	181 (8.1)	172 (95.0) [90.7-97.7]	9 (5.0) [2.3-9.3]	
October	164 (7.4)	146 (89.0) [83.2-93.3]	18 (11.0) [6.7-16.8]	
November	192 (8.6)	169 (88.0) [6.9-16.3]	23 (12.0) [7.8-17.5]	
December	160 (7.2)	141 (88.1) [82.0-92.7]	19 (11.9) [7.3-17.9]	

CI: Confidence Interval, P-value is significant at p < 0.050. MTB: *Mycobacterium tuberculosis*

The year-on-year prevalence of *Mycobacterium tuberculosis* decreased steadily from 10.7% in 2022 to 9.5% in 2023 and 7.2% in 2024. Adolescents (13–17 years) had significantly lower prevalence across all three years (*p* < 0.050). A marked difference was observed between sexes, with females consistently showing significantly higher *Mycobacterium tuberculosis* positivity than males each year: 72.6% vs. 6.0% in 2022 (*p* < 0.001), 12.9% vs. 5.9% in 2023 (*p* = 0.001), and 9.2% vs. 5.2% in 2024 (*p* = 0.033). Monthly distribution showed no consistent pattern, though higher prevalence rates were recorded in May, August and October, in 2022. Table 2.

**Table 2:** Year-on-year prevalence of *Mycobacterium tuberculosis.*

Variables	2022			2023			2024		P- value
	Total	MTB	P-value	Total	MTB	P- value	Total	MTB	
	n (%)	n (%)		n (%)	n (%)		n (%)	n (%)	
**Total**	681 (100.0)	73 (10.7)		797 (100.0)	76 (9.5)		747 (100.0)	54 (7.2)	
**Age group (years)**									
Infants (Under 1)	17 (2.5)	0 (0.0)		19 (2.4)	0 (0.0)		18 (2.4)	4 (22.2)	
Toddlers and pre-schoolers (1–4)	24 (3.5)	0 (0.0)		22 (2.8)	1 (4.6)		27 (3.6)	1 (3.7)	
School-age children (5–12)	51 (7.5)	1 (2.0)		36 (4.5)	1 (2.8)		65 (8.7)	1 (1.5)	
Adolescents (13–17)	27 (4.0)	1 (3.7)	**0.011**	32 (4.0)	0 (0.0)	**0.023**	32 (4.3)	1 (3.1)	**0.041**
Young adults (18–24)	43 (6.3)	7 (16.3)		60 (7.5)	10 (16.7)		53 (7.1)	3 (5.7)	
Adults (25–64)	403 (59.2)	55 (13.7)		488 (61.2)	55 (11.3)		432 (57.8)	38 (8.8)	
Elderly (65 and above)	116 (17.0)	9 (7.8)		140 (17.6)	9 (6.4)		120 (16.1)	6 (5.0)	
**Sex**									
Female	331 (48.6)	20 (6.0)		387 (48.6)	23 (5.9)		367 (49.1)	19 (5.2)	
Male	350 (51.4)	53 (72.6)	**<0.001**	410 (51.4)	53 (12.9)	**0.001**	380 (50.9)	35 (9.2)	**0.033**
**Months**									
January	47 (6.9)	4 (8.5)		90 (11.3)	11 (12.2)		68 (9.1)	10 (14.7)	
February	60 (8.8)	6 (10.0)		69 (8.7)	6 (8.7)		63 (8.4)	5 (7.9)	
March	61 (8.9)	5 (8.2)		86 (10.8)	7 (8.1)		49 (6.6)	2 (4.1)	
April	39 (5.7)	5 (12.8)		87 (10.9)	71 (8.9)		58 (7.8)	1 (1.7)	
May	62 (9.1)	9 (14.5)		71 (8.5)	6 (8.5)		60 (8.0)	5 (8.3)	
June	70 (10.3)	6 (8.6)	0.482	65 (8.2)	5 (7.7)	0.626	51 (6.8)	3 (5.9)	0.289
July	56 (8.2)	3 (5.4)		41 (5.1)	6 (14.6)		65 (8.7)	4 (6.2)	
August	68 (10.0)	10 (14.7)		69 (8.7)	5 (7.3)		73 (9.8)	5 (6.9)	
September	50 (7.3)	2 (4.0)		65 (8.2)	5 (7.7)		66 (8.8)	2 (3.0)	
October	62 (9.1)	11 (17.7)		48 (6.0)	4 (8.3)		54 (7.2)	3 (5.6)	
November	63 (9.3)	7 (11.1)		65 (8.2)	10 (15.4)		64 (8.6)	6 (9.4)	
December	43 (6.3)	5 (11.6)		41 (5.1)	6 (14.6)		76 (10.2)	8 (10.5)	

P-value is significant at p < 0.050.

Out of the 203 confirmed *Mycobacterium tuberculosis* cases, 166 (81.8%) underwent rifampicin susceptibility testing. Among these, 152 (91.6%) were susceptible, 11 (6.6%) showed intermediate resistance, and 3 (1.8%) were resistant. Although age was not significantly associated with rifampicin resistance (*p* = 0.068), adults aged 25–64 years constituted the majority of resistant cases (2.5%). Female patients had a higher proportion of resistance (4.1%) compared to males (0.9%), though the difference was not statistically significant (*p* = 0.166). Resistance patterns over the years showed a gradual increase, with 0.0% in 2022, 2.7% in 2023, and 5.6% in 2024, but this finding was also not statistically significant (*p* = 0.416). Monthly analysis revealed the highest resistance in December (14.3%) and intermediate resistance peaks in June and July (22.2%), although no significant monthly variation was observed (*p* = 0.146). Table 3.

**Table 3:** Prevalence and risk factors of rifampicin among presumptive cases.

Variables	Rifampicin				
	Overall	Susceptible	Intermediate	Resistant	P-value
	n (%)	n (%)	n (%)	n (%)	
Total	166 (100.0)	152 (91.6)	11 (6.6)	3 (1.8)	
**Age (years)**					
Infants (Under 1)	3 (1.8)	1 (33.3)	2 (66.7)	0 (0.0)	
Toddlers and pre-schoolers (1–4)	1 (0.6)	1 (100.0)	0 (0.0)	0 (0.0)	
School-age children (5–12)	3 (1.8)	3 (100.0)	0 (0.0)	0 (0.0)	
Adolescents (13–17)	1 (0.6)	1 (100.0)	0 (0.0)	0(0.0)	0.068
Young adults (18–24)	18 (10.8)	17 (94.4)	1 (5.6)	0 (0.0)	
Adults (25–64)	121 (72.9)	112 (92.5)	6 (5.0)	3 (2.5)	
Elderly (65 and above)	19 (11.5)	17 (89.5)	2 (10.5)	0 (0.0)	
**Sex**					
Female	49 (29.5)	42 (85.7)	5 (10.2)	2 (4.1)	
Male	117 (70.5)	110 (94.0)	6 (5.1)	1 (0.9)	0.166
**Year**					
2022	73 (44.0)	69 (94.5)	4 (5.5)	0 (0.0)	
2023	75 (45.2)	68 (90.7)	5 (6.7)	2 (2.7)	0.416
2024	18 (10.8)	15 (83.3)	2 (11.1)	1 (5.6)	
**Months**					
January	25 (15.1)	21 (84.0)	3 (12.0)	1 (4.0)	
February	16 (9.6)	15 (93.8)	1 (6.2)	0 (0.0)	
March	13 (7.8)	13 (100.0)	0 (0.0)	0 (0.0)	
April	12 (7.2)	12 (100.0)	0 (0.0)	0 (0.0)	
May	15 (9.0)	14 (93.3)	1 (6.7)	0 (0.0)	
June	9 (5.4)	7 (77.8)	2 (22.2)	0 (0.0)	0.146
July	9 (5.4)	7 (77.8)	2 (22.2)	0 (0.0)	
August	15 (9.0)	15 (100.0)	0 (0.0)	0 (0.0)	
September	7 (4.2)	6 (85.7)	1 (14.3)	0 (0.0)	
October	15 (9.0)	15 (100.0)	0 (0.0)	0 (0.0)	
November	16 (9.4)	15 (93.8)	1 (6.3)	0 (0.0)	
December	14 (8.4)	12 (85.7)	0 (0.0)	2 (14.3)	

P-value is significant at p < 0.050.

**Fig 1:**
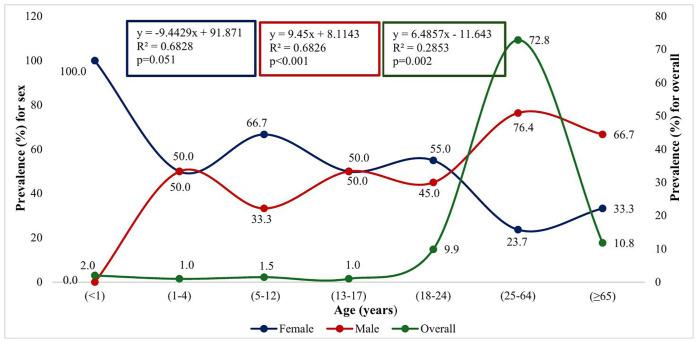
Distribution of tuberculosis across age groups stratified by sex. The overall tuberculosis prevalence shows considerable monthly variation, peaking in January (12.3%) and November (11.3%), while reaching its lowest point in September (4.4%). Male tuberculosis cases demonstrate a bimodal distribution with peaks in February (82.4%) and September (100%), suggesting potential seasonal clustering, whereas female cases show an inverse relationship, with the highest representation in January (48%) and July (46.2%) and complete absence in September (0%). Fig 2.

Fig 1 shows the age-specific prevalence of *Mycobacterium tuberculosis* cases stratified by sex and overall distribution. The prevalence among females showed a decreasing rate with increasing age (R² = 0.6828, *p* = 0.051), while males demonstrated a significant increase (R² = 0.6826, *p* < 0.001). Overall prevalence also increased significantly with age, peaking in the 25–64 age group (72.8%) before declining in those aged ≥65 years (R² = 0.2853, *p* = 0.002). The R² value, which represents the proportion of variance explained by age in each model, indicates a strong association for both sexes (R² = 68%) and a moderate association for the overall prevalence (R² = 28.5%). 100% of cases in infants were female, while males predominated in the adult age groups, particularly 25–64 years (76.4%).

**Fig 2:**
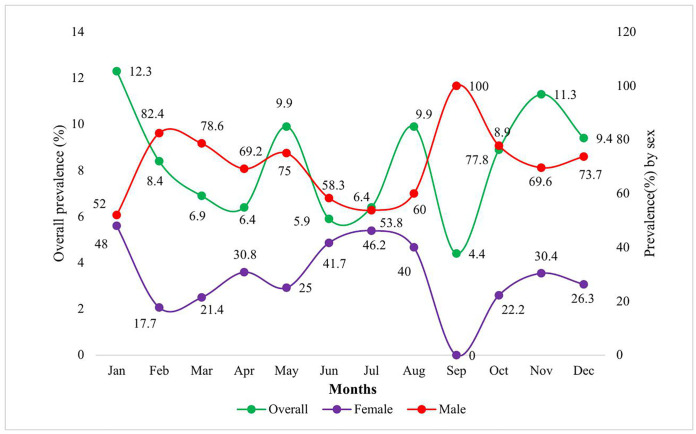
Monthly distribution of tuberculosis stratified by sex. In Model 2, males had significantly higher odds and prevalence of tuberculosis compared to females (aOR: 2.44; aPR: 2.21; *p* < 0.001). Compared to infants, school-age children (5–12 years) showed a significantly lower likelihood of tuberculosis (aOR: 0.20; *p* = 0.044). The year 2024 and September were also associated with reduced tuberculosis risk relative to 2022 (aOR: 0.63; *p* = 0.018) and January (aOR: 0.40; *p* = 0.024), respectively. Model 2 demonstrated superior performance over Model 1, with lower AIC (1309.96 vs. 1340.46), lower BIC (1429.82 vs. 1454.61), and a higher AUC (0.7025 vs. 0.6576). Table 4.

**Table 4:** Adjusted prevalence ratio and adjusted odds ratio of factors associated with tuberculosis.

Variables	Model 1		Model 2	
	aPR [95% CI] P-value	aOR [95% CI] P-value	aPR [95% CI] P-value	aOR [95% CI] P-value
**Sex**				
Female			1	1
Male			2.21 [1.66-2.95] <0.001	2.44 [1.77-3.34] <0.001
**Age group, years**				
Infants (under 1)	1	1	1	1
Toddlers and pre-schoolers (1–4)	0.26 [0.07-1.86] 0.220	0.33 [0.06-1.89] 0.214	0.33 [0.06-1.76] 0.195	0.29 [0.05-1.69] 0.170
School-age children (5–12)	0.26 [0.06-1.13] 0.072	0.24 [0.05-1.13] 0.071	0.23 [0.05-1.02] 0.053	0.20 [0.04-0.96] **0.044**
Adolescents (13–17)	0.29 [0.06-1.55] 0.148	0.27 [0.05-1.56] 0.144	0.26 [0.05-1.38] 0.114	0.23 [0.04-1.30] 0.097
Young adults (18–24)	1.71 [0.61-4.77] 0.309	1.81 [0.58-5.58] 0.304	1.65 [0.58-4.67] 0.348	1.74 [0.56-5.41] 0.341
Adults (25–64)	1.47 [0.56-3.82] 0.434	1.51 [0.53-4.27] 0.436	1.36 [0.51-3.59] 0.538	1.33 [0.47-3.79] 0.592
Elderly (65 and above)	0.85 [0.31-2.36] 0.756	0.83 [0.27-2.51] 0.743	0.78 [0.28-2.20] 0.640	0.72 [0.24-2.20] 0.568
**Years**				
2022	1	1	1	1
2023	0.86 [0.63-1.17] 0.331	0.84 [0.60-1.20] 0.341	0.84 [0.62-1.13] 0.256	0.83 [0.58-1.17] 0.286
2024	0.67 [0.48-0.93] **0.017**	0.64 [0.44-0.93] **0.018**	0.67 [0.48-0.93] **0.016**	0.63 [0.43-0.93] **0.018**
**Month**				
January	1	1	1	1
February	0.79 [0.44-1.40] 0.414	0.75 [0.39-1.44] 0.384	0.83 [0.47-1.48] 0.539	0.77 [0.40-1.50] 0.443
March	0.62 [0.33-1.14] 0.124	0.56 [0.28-1.13] 0.106	0.63 [0.34-1.17] 0.145	0.56 [0.28-1.12] 0.103
April	0.61 [0.32-1.15] 0.126	0.56 [0.28-1.14] 0.113	0.70 [0.37-1.32] 0.266	0.64 [0.31-1.30] 0.215
May	0.88 [0.51-1.52] 0.643	0.85 [0.45-1.60] 0.613	0.99 [0.57-1.70] 0.958	0.94 [0.50-1.78] 0.844
June	0.54 [0.28-1.05] 0.070	0.51 [0.25-1.05] 0.066	0.58 [0.30-1.13] 0.110	0.53 [0.26-1.11] 0.093
July	0.70 [0.37-1.33] 0.275	0.67 [0.33-1.37] 0.276	0.78 [0.41-1.49] 0.455	0.74 [0.36-1.53] 0.419
August	0.80 [0.46-1.40] 0.436	0.76 [0.41-1.44] 0.404	0.90 [0.52-1.57] 0.719	0.85 [0.45-1.60] 0.613
September	0.43 [0.21-0.90] **0.024**	0.39 [0.18-0.86] **0.020**	0.46 [0.22-0.95] **0.037**	0.40 [0.18-0.89] **0.024**
October	0.94 [0.53-1.66] 0.833	0.91 [0.47-1.75] 0.777	0.95 [0.54-1.67] 0.864	0.90 [0.46-1.74] 0.748
November	1.02 [0.60-1.73] 0.944	1.01 [0.55-1.86] 0.983	1.13 [0.67-1.91] 0.647	1.10 [0.60-2.05] 0.752
December	1.12 [0.64-1.95] 0.697	1.11 [0.58-2.12] 0.750	1.16 [0.67-2.02] 0.593	1.14 [0.59-2.20] 0.690
**Model significance**		<0.001		<0.001
**Information criteria**				
Akaike information criteria (AIC)		1340.46		1309.96
Bayesian information criteria (BIC)		1454.61		1429.82

CI: Confidence Interval, aOR: Adjusted odds ratio, aPR: Adjusted prevalence ratio. P-value is significant at p < 0.050.

**Fig 3:**
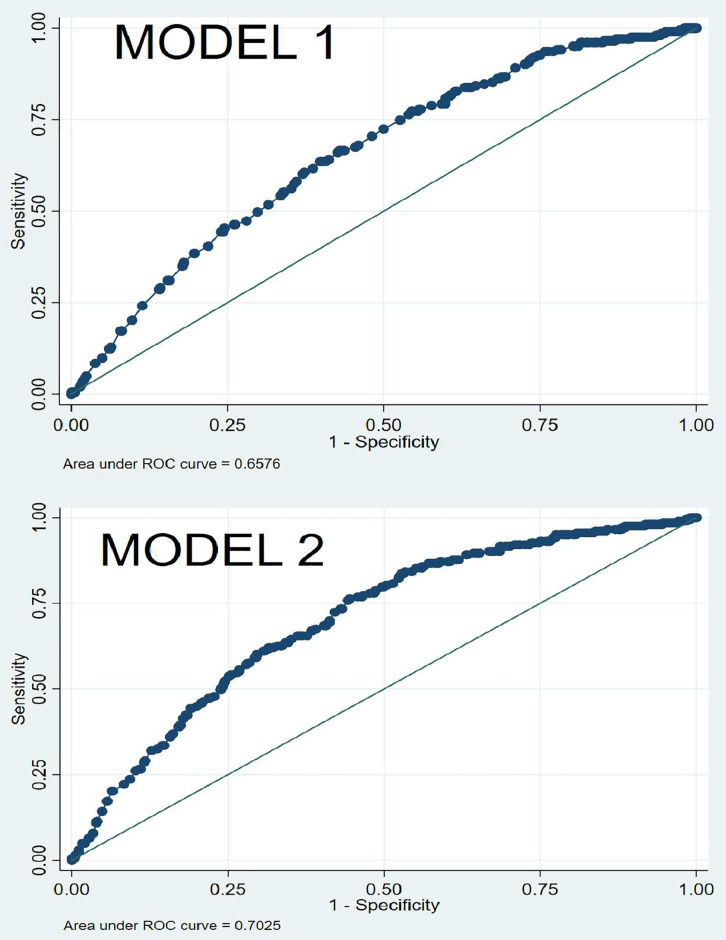
ROC curve of the binary logistic regression model.

The Receiver Operating Characteristics (ROC) curve analysis demonstrated that Model 2, which adjusted for age, year, sex, and month, exhibited improved discriminatory performance in identifying factors associated with tuberculosis compared to Model 1, which adjusted for age, year, and month only. Model 2 achieved an Area Under the ROC Curve (AUC) of 0.7025, indicating fair predictive accuracy, while Model 1 recorded a lower AUC of 0.6576. The inclusion of sex as an additional covariate in Model 2 appears to enhance model performance, suggesting that sex may be a relevant factor in predicting tuberculosis risk in this population. Fig 3.

## Discussion

This current study in the Ho Teaching Hospital reports findings on the prevalence and risk factors associated with *Mycobacterium tuberculosis* and rifampicin-resistant tuberculosis (RR-TB) in a large cohort of presumptive tuberculosis cases over a three-year period. Among the 2,225 presumptive cases analysed, the overall prevalence of infection was 9.1%, with distinct variations across age, sex, and time. This prevalence is slightly lower (10.76%) than that reported by Deku, Aninagyei [6] at the Ho Teaching Hospital, Ghana, but still suggests a considerable disease burden that warrants strengthened diagnostic and control efforts. Elsewhere in Gedeo Zone, Southern Ethiopia, an overall prevalence of *Mycobacterium tuberculosis* was 26.8% in a study by Diriba and Churiso [9]. This is significantly higher than the prevalence reported in this study. Also in Eastern Amhara, Ethiopia, Wasihun, Hailu [10] reported the prevalence of the infection to be 11% which is higher than what has been recorded in this study. In addition to the high prevalence of the infection reported in a previous study compared to this current study, Ugwu, Agbo [11] found 22.1% *Mycobacterium tuberculosis* prevalence among 868 study participants in Nigeria. This finding emphasises that tuberculosis remains a leading infectious disease cause of death, with about 1.25 million deaths in 2023. The disease disproportionately affects developing countries, with over 95% of deaths occurring there [12].

The age group with the highest *Mycobacterium tuberculosis* prevalence was young adults aged 18–24 years (12.8%), followed closely by adults aged 25–64 years (11.2%). These findings align with previous research in Ghana by Deku, Aninagyei [6] and Zambia by Kapata, Chanda-Kapata [13], which consistently report increased tuberculosis burden among economically productive adults. The relatively low prevalence among children under 12 years may reflect reduced exposure to active tuberculosis sources or more effective tuberculosis control in paediatric settings. However, underdiagnosis in children due to challenges in sputum sample collection cannot be ruled out.

The study also revealed a significant sex disparity in infection prevalence, with males exhibiting more than twice the burden observed in females (12.4% vs 5.7%). The aOR of 2.44 confirmed that male sex was a strong predictor of the infection. This observation is consistent with studies in Ghana, Ethiopia, and other low- and middle-income countries [6,14–17] and may be attributed to gender-related differences in health-seeking behaviours, occupational exposure, and immunological responses.

Regarding yearly distribution, the prevalence of the infection decreased from 10.7% in 2022 to 7.2% in 2024. This downward rate may indicate the positive impact of enhanced tuberculosis interventions, although it must be interpreted with caution, given the fluctuating monthly prevalence and potential diagnostic or reporting inconsistencies. Interestingly, while the annual prevalence suggests improvement, certain months (January, May, October, and December) showed increased case detection, possibly pointing to seasonal or behavioural influences on transmission. This contrasts slightly with the Ho Teaching Hospital study by Deku, Aninagyei [6], where rainy seasons were associated with higher infection prevalence.

The detection of rifampicin resistance among newly diagnosed *Mycobacterium tuberculosis* cases, though relatively low (1.8%), is of public health concern. In other parts of Ghana, Boakye-Appiah, Steinmetz [18] and Sylverken, Kwarteng [19] reported rifampicin-resistant *Mycobacterium tuberculosis* rates of 14.4% and 2.4% respectively. In a related study conducted in selected government hospital in Addis Ababa, Ethiopia, Arega, Menbere [20] found the prevalence of rifampicin-resistant *Mycobacterium tuberculosis* among presumptive tuberculosis patients to be 9.9%. This is significantly higher than recorded in this study. The rising rifampicin resistance among newly diagnosed *Mycobacterium tuberculosis* cases, increasing from 0.0% in 2022 to 5.6% in 2024, is concerning. Contributing factors may include poor treatment adherence, delays in diagnosis and initiation of therapy, irregular or interrupted drug supply, and circulation of resistant strains within communities and calls for urgent reinforcement of drug-resistance surveillance and early treatment initiation in Ghana’s National TB Control Strategy. Females demonstrated a slightly higher resistance rate (4.1%) than males (0.9%), though the difference was not statistically significant. This finding corroborated a previous study [6], where rifampicin-resistant *Mycobacterium tuberculosis* was slightly more prevalent in females, but still not statistically significant. The highest resistance burden was seen in adults aged 25–64 years, which aligns with their higher infection prevalence.

In summary, this current study adds that tuberculosis remains a pressing health issue among presumptive cases, especially among males and adults aged 18–64. The increasing rate of rifampicin resistance, though limited in numbers, is alarming and warrants continuous monitoring. The differences seen between sexes and seasons show the need for targeted actions. Seasonal changes may be due to weather conditions or health-seeking habits, like less hospital visits during the rainy season or more indoor crowding in colder months, which can increase TB spread.

### Limitations of the study

The retrospective design of the study did not allow the authors to compare the laboratory findings to the patients’  clinical presentations.

## Conclusion

The prevalence of MTB was 9.1%, highest among adults aged 18–24 years (12.8%), while rifampicin resistance, though low (1.8%), remains a public health concern. Expanding GeneXpert coverage, strengthening drug-resistance monitoring, and targeting high-risk groups, especially adult males, are critical steps that align with Sustainable Development Goal 3 to end TB by 2030.

## Supporting information

S1 Data Tuberculosis Ho Teaching Hospital data. (XLSX)
